# To Regulate the Practice in All the States

**Published:** 1887-11

**Authors:** 


### TO REGULATE THE PRACTICE IN ALL THE STATES.

It is, perhaps, not generally known, that at the June '87 meeting of the American Medical Association, a resolution was adopted appointing a committee of five, to report at the first meeting of the Section or State Medicine next year, to frame a bill, for recommandation to different States, regulating the practice of medicine. This committee consists of Dr. P. Millard and Dr. H. A. Johnson, of Chicago, Dr. Geo. B. Belt, of Mass., and Dr. C. W. Dulles, of Pennsylvania.

Our committee should communicate with them.

*Apropos.* Dr. Frank H. Tucker, of San Augustine, Texas, has been appointed on the committee of the Texas State Medical Association, entrusted with work in this department, in place of Dr. C. H. Wilkinson, who was excused by President Buroughs.

